# Adult vector control, mosquito ecology and malaria transmission

**DOI:** 10.1093/inthealth/ihv010

**Published:** 2015-02-26

**Authors:** Oliver J. Brady, H. Charles J. Godfray, Andrew J. Tatem, Peter W. Gething, Justin M. Cohen, F. Ellis McKenzie, T. Alex Perkins, Robert C. Reiner, Lucy S. Tusting, Thomas W. Scott, Steven W. Lindsay, Simon I. Hay, David L. Smith

**Affiliations:** a Spatial Ecology and Epidemiology Group, Department of Zoology, Oxford University, Oxford, UK; b Department of Zoology, University of Oxford, UK; c Department of Geography and Environment, University of Southampton, Southampton, UK; d Fogarty International Center, NIH, Bethesda, MD, USA; e Flowminder Foundation, Stockholm, Sweden; f Clinton Health Access Initiative, Boston, MA, USA; g Department of Biological Sciences and Eck Institute for Global Health, University of Notre Dame, Notre Dame, IN, USA; h Department of Entomology and Nematology, University of California, Davis, CA, USA; i Department of Epidemiology and Biostatistics, Indiana University, Bloomington, IN, USA; j Department of Disease Control, London School of Hygiene & Tropical Medicine, London, UK; k School of Biological Sciences, Durham University, Durham, UK; l Sanaria Institute for Global Health and Tropical Medicine, Rockville, MD, USA

**Keywords:** Larval control, Malaria control policy, Micro-simulation models, *Plasmodium falciparum*, *Plasmodium vivax*, Vectorial capacity

## Abstract

**Background:**

Standard advice regarding vector control is to prefer interventions that reduce the lifespan of adult mosquitoes. The basis for this advice is a decades-old sensitivity analysis of ‘vectorial capacity’, a concept relevant for most malaria transmission models and based solely on adult mosquito population dynamics. Recent advances in micro-simulation models offer an opportunity to expand the theory of vectorial capacity to include both adult and juvenile mosquito stages in the model.

**Methods:**

In this study we revisit arguments about transmission and its sensitivity to mosquito bionomic parameters using an elasticity analysis of developed formulations of vectorial capacity.

**Results:**

We show that reducing adult survival has effects on both adult and juvenile population size, which are significant for transmission and not accounted for in traditional formulations of vectorial capacity. The elasticity of these effects is dependent on various mosquito population parameters, which we explore. Overall, control is most sensitive to methods that affect adult mosquito mortality rates, followed by blood feeding frequency, human blood feeding habit, and lastly, to adult mosquito population density.

**Conclusions:**

These results emphasise more strongly than ever the sensitivity of transmission to adult mosquito mortality, but also suggest the high potential of combinations of interventions including larval source management. This must be done with caution, however, as policy requires a more careful consideration of costs, operational difficulties and policy goals in relation to baseline transmission.

## Introduction

Vector-based interventions have been and continue to be a major component of programs aiming to reduce the public health burden of mosquito-borne diseases. Over the past eleven decades the dominant methods recommended for vector control have shifted from larval source management (LSM), house screening and bednets,^1^ to indoor residual spraying with effective contact pesticides,^2^ and in recent years to long-lasting insecticidal nets, as funding increased and intervention coverage levels were scaled up to reach the Millennium Development Goals for malaria.^3^ New vector control methods are being developed and tested at an unprecedented rate,^4^ including genetically modified mosquitoes,^5^ oviposition traps,^6^ spatial repellents^7^ and attractive toxic nectar baits.^8^ The availability of so many methods raises questions about how to use these methods optimally alone and in combination in different contexts to achieve policy goals. These contexts may differ with respect to vector species, habitats and ecologies, sociological settings and control regimes. Specifically, it is realised that the elimination of vector-borne diseases is unlikely to be achieved using a single method and that multiple interventions are required that are adapted to local conditions, a concept recognised by the World Health Organization in its support for integrated vector management.^9^ A simple mathematical analysis describing the sensitivity of transmission to adult mosquito longevity has played a major historical role in guiding policy,^10–12^ and mathematical models in general, particularly newly developed micro-simulation models, have been used to provide a basis for evaluating vector transmission dynamics and integrated control.^13–15^ In this study, we update the mathematical theory supporting malaria control in light of several models published recently that now allow a consideration of both adult and juvenile mosquito stages and their relation to malaria transmission.

Mathematical models provide tools for thinking carefully and quantitatively about malaria and other mosquito-borne pathogens.^16,17^ While they may not exactly replicate every detail of reality in every setting, their general insights are indispensable to the investigation of complex topics in science and for turning scientific knowledge into policy. Most current mathematical models of malaria are derived from a simple model developed by Ronald Ross and George Macdonald,^18,19^ including formulae describing the basic reproductive number for malaria, *R*_0_, from which the concept of vectorial capacity was derived.^10,18,20,21^ Because the vast majority of mathematical models describing pathogen transmission by mosquitoes make the same assumptions as Macdonald's model,^19^ understanding vectorial capacity remains relevant today and can provide insight into many problems faced by contemporary policy debates (Box 1).

Box 1. **Policy Interpretation**

With a wide variety of vector control methods now available policy makers are increasingly interested in asking, ‘Which methods are most effective?’ and, ‘How can they be combined to reach policy goals in different transmission settings?’

Mathematical models of transmission provide insight about these questions. Historically the most frequently used models have omitted descriptions of the juvenile mosquito life stages and have thus not been able to evaluate the effectiveness of interventions such as larval source management (LSM). While modern digital methods in epidemiology, namely individual-based micro-simulations, often do describe these aspects of mosquito life history adequately, more basic theory that is often used to steer policy have not accounted for these factors to date.

In this analysis we develop the framework of vectorial capacity to include this important omission. This allows us to revisit arguments about why adult killing methods have been preferred since the formation of the Global Malaria Eradication Programme. Our results showed that while adult killing methods are likely to affect transmission by an even greater magnitude than previously thought, combination with other methods such as LSM is likely to be important under some circumstances. This is particularly true where mosquito populations are vulnerable to elimination.

These results must be interpreted in light of additional operational considerations of cost-effectiveness of different interventions and the coverage levels that can be reached in different transmission settings.

Macdonald's original mathematical parameter sensitivity analysis suggested the intensity of transmission by mosquitoes would be highly sensitive to the lifespan of adult female mosquitoes.^10,22^ New insights came from re-examining that model using realistic assumptions about adult mosquito population compensation, in which Smith and McKenzie^22^ showed that reducing adult lifespan would also reduce adult mosquito population density. More recently, new simulation models have been developed that consider the feedbacks between egg laying by adults, maturation and survival in heterogeneous aquatic habitats, and emergence of juveniles into adults.^13,15,23,24^ Simulation models such as these expand on the older theory and, unlike the classical models, describe a mathematical basis for understanding and evaluating LSM.^13,23^ These models also establish a more comprehensive way of understanding the wider effects of adult vector control. This developed understanding of mosquito population dynamics has yet to be incorporated into the most commonly used descriptions of control using vectorial capacity.

Analytical and simulation analyses offer two different ways of understanding how processes operate in a given mathematical model. The former enables mathematical manipulation of the initial model to derive directly interpretable submodels for component processes. This enables an interpretation of how generalizable a process may be, or how it might change in certain situations. Deriving analytical solutions to models generally becomes harder as models become more complex. By contrast, simulation analyses evaluate model processes using predefined sets of parameters representing specific situations. Simulation analyses can be performed on very complex models, but the general question remains how robust are the resulting processes to changes in the underlying parameters? This can be tested with a parameter sensitivity analysis, but it remains unclear whether the phenomenon applies to the general case as not every possible combination of parameters are tested. We can be more confident about which processes are most important for transmission if the same trends appear in analytical analyses of simpler, more generalizable transmission models and in simulation analyses of specialized complex transmission models.

In this study we analyse a mathematical model to explore how adult vector control could be expected to modify mosquito population density. We use analytical approaches to examine mosquito population responses under various assumptions about migration and homogeneity, then use simulation approaches to test the response in more realistic heterogeneous, open conditions. Inferences from both of these approaches allow us to update the mathematical theory describing the expected relationship between vector control strategies and vectorial capacity.

## Materials and methods

### Mathematical model

We develop the model from Smith et al.^13^ by including migration dynamics in order to consider mosquito population dynamics, vector ecology and changes in mosquito density within a circumscribed area. The model considers changes in adult and juvenile population densities through two coupled ordinary differential equations. Let *m* denote the ratio of adult female mosquitoes to humans in the area. We assume the human population size is constant, therefore changes in *m* reflect changes in adult mosquito density. Let *δ* denote the number of adult mosquitoes entering the population from outside the area, per human, per day and let *ω* denote the rate that mosquitoes exit from the area. Let *g* denote the per-capita death rate of adult mosquitoes, *f* the per-mosquito blood feeding rate (on any hosts), and *v* the number of female eggs laid by a female mosquito per bloodmeal. Aquatic habitats in this model are subdivided into *N* distinct habitats, termed ‘pools’ in this case for the sake of simplicity, and *l_i_* is the number of juveniles in the *i* th pool. Juvenile mosquitoes transition from juveniles to adults (i.e. mature) at a pool-specific, constant per-capita rate, *a_i_*, and die at the pool-specific, per-capital rate $\gamma_{i}+\psi_{i}l_{i}^{\sigma_{i}}$. These death rates can be considered in two parts: the family of factors summarised by parameter *γ_i_* describes all sources of density independent mortality, and the power-law function $\psi_{i}l_{i}^{\sigma_{i}}$ describes mortality rates as a function of mean density. When $\sigma_{i}=1$, as it does throughout this analysis, the model is an analogue of the logistic growth equations for the low levels of *l_i_* representative of most field populations and is supported by experimental studies on density dependence in larval habitats.^25^ This assumes no age structure or stage divisions of the juvenile cohorts and therefore can only evaluate population responses to mean densities. We also assume that increased density has no adverse effects on the emerging adults. When evaluated with a small number of pools, such as the *N*=30 used in this analysis, the model is representative of mosquito population dynamics at a village or neighbourhood level where each pool may differ in attractiveness, larval resources and sources of biotic and abiotic mortality. The final step links the proportion of eggs laid in each habitat to the adult mosquito population, *j_i_* . The resulting system has *N*+1 coupled ordinary differential equations describing adult:

$$\frac{dm}{dt}=\overset{N}{\underset{i=1}{\sum }}\alpha_{i}l_{i}-(g+\omega )m+\delta ,$$

and juvenile population dynamics:

$$\frac{dl_{i}}{dt}=fvj_{i}m-(\alpha_{i}+\gamma_{i}+\psi_{i}l_{i}^{\sigma_{i}})l_{i}.$$

The focus of the analysis described herein is change in adult female mosquito density and vectorial capacity at the steady state; i.e. looking at properties of solutions to the equations where $dm\text{/}dt=dl_{i}\text{/}dt=0.$ A full list of parameters used in the manuscript and their explanation is given in Table 1.

**Table 1.** Parameters and other terms from various formulae for vectorial capacity used in this paper. Where no units are given, the units are pure numbers

Symbol	Alternatives	Units	Short name	Explanations
*m*	*λ/g*	NA	Mosquitoes per human	Ratio of mosquito population density to human population density
*p*	$e^{-g}$	*d*	Daily survival	The probability a mosquito survives one day
*n*	NA	*d*	EIP	Average no. of days between mosquito infection and the appearance of sporozoites in the salivary glands
*a*	*fQ*	*d*^−1^	Human blood feeding rate	Average number of human blood meals, per mosquito, per day
*f*	NA	*d*^−1^	Blood feeding rate	Average number of blood meals, per mosquito, per day
*Q*	NA	NA	Human feeding propensity	Average proportion of blood meals taken on humans
*g*	NA	*d*^−1^	Mosquito mortality rate	Mosquito per-capita daily mortality rate
*λ*	NA	*d*^−1^	Adult female mosquito emergence rate	No. of adult female mosquitoes emerging from aquatic habitat, per human, per day
*δ*	NA	*d*^-1^	Mosquito immigration rate	No. of adult female mosquitoes entering the population, per human, per day
*ω*	NA	*d*^-1^	Mosquito emigration rate	No. of adult female mosquitoes leaving the population, per human, per day
*S*	*fQ*/*g*	NA	Stability index	No. of human blood meals taken by a mosquito summed over its entire lifespan
*P*	$p^{n}=e^{-gn}$	NA	EIP Survival	Proportion of mosquitoes that survive EIP
*v*	NA	NA	Female eggs batch size	No. of female eggs laid by a female mosquito each time it oviposits
*G*	*vf*/*g*	NA	Lifetime female eggs laid	No. of female eggs laid by a female mosquito summed over its lifespan
*γ*	NA	*d*^-1^	Pool fixed mortality rate	Juvenile mosquito per-capita daily density independent mortality rate
$\psi_{i}l_{i}^{\sigma_{i}}$	NA	*d*^-1^	Pool density dependent mortality rate	Juvenile mosquito per-capita daily density dependent mortality rate
*j_i_*	NA	NA	Egg laying proportion	The proportion of total eggs of an adult female laid in the *i* th pool

EIP: extrinsic incubation period; NA: not applicable.

### Effect sizes and elasticity analysis

We extend Macdonald's analysis using the concept of effect sizes (*E_C_*),^26^ which are proportional reductions in transmission brought about by proportional changes in mosquito bionomic parameters in response to vector control, and described in a formula by the ratio of baseline vectorial capacity (*V*_0_) to its value with vector control (*V_C_*) denoted: $E_{C}=V_{0}/V_{C}.$

The relevance of changes in adult mosquito bionomic parameters affecting vectorial capacity is examined by looking at effect sizes associated with changes in some parameter. The effect size associated with changes in a parameter *x* is defined by its baseline *x*_0_ and its new value under control *x*_C_:

$$E_{V}(x_{C}|x_{0})=\frac{V(x_{0})}{V(x_{C})}.$$

The effect sizes associated with large changes in *x* can be evaluated using the whole effect size function, but some useful insights come from a sensitivity analysis, which looks at the changes in *E* associated with small changes in *x* around the baseline:

$$\left(\frac{dE_{V}(x_{C}|x_{0})}{dx}|x=x_{0}\right)=-\frac{V^{\prime }(x_{0})}{V(x_{0})}.$$

Since an effect size is defined as a proportional change in transmission, it is of greater interest to look at the elasticity, a measure that compares effect sizes for small proportional changes in *x* around baseline, which is defined by the following:

$$\epsilon (x_{0})=\left(\frac{dE_{V}(\theta x_{0}|x_{0})}{d\theta }|\theta =1\right)=-x_{0}\frac{V^{\prime }(x_{0})}{V(x_{0})}.$$

Three simple rules make it trivial to compute the elasticities of the parameters and functions in any formula for vectorial capacity that does not explicitly consider the effects of mosquito population dynamics: 1) If $V(x)=bx^{k}$, where *b* is any constant, then ϵ(x)=−k; 2) If $V(x)=be^{-xy}$, then $\epsilon_{E}(x)=xy$, so the elasticity of *x* depends on *y*; 3) Elasticities are additive, for if V(x)=f(x)g(x), then $\epsilon_{V}(x_{0})=\epsilon_{f}(x_{0})+\epsilon_{g}(x_{0})$.

## Results

The following analysis updates and repeats Macdonald's classical analysis for an expanded formula for vectorial capacity including feedbacks between adult and juvenile mosquito populations. This analysis looks at the effects on vectorial capacity of proportionally changing three adult bionomic parameters (*f*, *v* and *g*).

### Vectorial capacity

The classical formula for vectorial capacity includes two parameters, in addition to those already defined above, that are required for transmission but not for mosquito population dynamics. These are the parasite's extrinsic incubation period (EIP, *n* days), and the proportion of blood meals taken on humans (*Q*).^21,27^ The original formula contained a single parameter to describe human blood feeding rates (alternatively human biting rate), *a*=*fQ*, and it used daily survival probability,$\;p=e^{-g}$. Note that lifespan of a mosquito is 1/g=1/−ln(p), therefore the formula was:

$$V=\frac{ma^{2}p^{n}}{-\text{ln}(\;p)}.$$

For our purposes it is useful to reformulate vectorial capacity to include both adult and juvenile population dynamic feedbacks. For this we must give a name to the net productivity of all the mosquito inhabited aquatic habitats; the number of adult mosquitoes emerging per human:

$$\lambda =\underset{i}{\sum }\alpha_{i}l_{i}.$$

Mosquito population density here is affected by immigration (*δ*), as well as internal recruitment, and the local population density depends on mortality as well as emigration (*ω*):

$$m=\frac{\lambda +\delta }{g+\omega }.$$

The ratio λ/δ is thus a useful measure of the relative importance of internal local mosquito dynamics, compared to the global effects of external populations

To look at the feedbacks from adult mosquito populations to juvenile aquatic populations, and vice versa, it is useful to define the number of eggs laid over their mosquito lifespan. Blood meals provision mosquito eggs, such that the number of blood meals is linked to the number of female eggs laid over an adult mosquito lifespan (G=vf/g), assuming blood meals are equally nutritious. The effects of egg laying on adult mosquito productivity are a priori non-linear, depending on the threshold condition for mosquito population persistence and the form of density-dependence.^13,15,23^

The modified formula for vectorial capacity we will examine here is:

$$V=\left(\lambda \left(\frac{vf}{g}\right)+\delta \right)\frac{\;f^{2}Q^{2}e^{-gn}}{g^{2}}=(\lambda (G)+\delta )\frac{\;f^{2}Q^{2}e^{-gn}}{g^{2}},$$

describing all the infectious bites that would arise anywhere from all the mosquitoes feeding on a single perfectly infectious human on a single day in the target population. Here mosquito immigration contributes to local population densities by adding to the adults emerging from aquatic juvenile populations. For assessing policy outcomes it may be more common to just assess reduction in transmission in the target area and ignore the effects on surrounding areas, in which case the following formula applies:

$$V=\left(\lambda \left(\frac{vf}{g}\right)+\delta \right)\frac{\;f^{2}Q^{2}e^{-(g+\omega )n}}{(g+\omega {)}^{2}}=(\lambda (G)+\delta )\frac{\;f^{2}Q^{2}e^{-(g+\omega )n}}{(g+\omega {)}^{2}}.$$

In the second formula, emigration (*ω*) affects the residence time in this population in the same way as death, though the formula implicitly assumes that had a mosquito flown out of the target area, it would not return. In this analysis we do not fully consider the feedbacks between local population dynamics and the surrounding environment, although the same framework could consider this if integrated with a metapopulation model.

One useful way to think about the terms describing immigration and emigration is the spatial scale of the population being considered. The larger the area, the more its population dynamics will be determined by local processes, and the less it will tend to be affected by migration. The ratio λ/δ can be thought of as a measure of the relative importance of local endogenous population dynamics to those in surrounding populations and/or spatial scale that is functionally relevant, from the perspective of the assumptions made about mosquito population dynamics in this model.

### Mathematical sensitivity and vector control

Elasticity analysis emphasises the mathematical order of the parameters. Changes in vectorial capacity are linearly proportional to changes in mosquito density (i.e. to *m* or *λ*): such effects are called 1^st^ order, a fact that is obvious from inspection because the parameters appear by themselves (i.e. not in an exponent) and only once. Similarly, those terms that appear twice have a 2^nd^ order effect (*f* and *Q*).

The term *n*, the EIP, also appears once in an exponent, where it is paired with *g*. The mathematical order of its elasticity is *ng*, a term that describes EIP as a fraction of mosquito lifespan. Because they always appear together, the order of the elasticity of *n* depends on the value of *g*: if EIP were on the order of mosquito lifespan (i.e.n≈1/g), then the elasticity of changing EIP would be approximately first order. If EIP were half of mosquito lifespan, then the elasticity would be of order ½, scaling as a square root. If EIP were twice as long as mosquito lifespan, elasticity would be quadratic, of order 2. Consideration of *n* illustrates why elasticity analysis is only valid for understanding small changes in effect sizes: for ‘large’ changes in *n* (or *g*, see below), the order of the elasticity grows linearly with proportional changes in *n*.

The original formula for vectorial capacity using Macdonald's notation (with $p=e^{-g}$), assumed that decreasing mosquito survival would not reduce mosquito population density, so the order of the effect was 1+ng. A model consistent with Macdonald's original assumptions is ‘perfect compensation’,^15^ where productivity of juvenile habitats increases when adult populations decrease to balance population losses and exactly compensate for adult mosquito mortality, but perfect compensation is mathematically complicated and biologically incompatible with observed patterns of juvenile positive density dependent mortality.^25^

A simpler alternative, closely related to the one described above, assumes constant productivity of aquatic habitats and tracks adults from the moment of emergence (i.e. formulated with a constant parameter *λ*, Table 2).^13^ In this model, mosquito survival has a linear effect on mosquito density, so the elasticity of mosquito lifespan on vectorial capacity is of order 2+*ng*. These effects correspond to a reduction in 1) the proportion of mosquitoes that ever become infected; 2) in the probability of surviving the EIP; 3) in the number of infectious bites.

**Table 2.** Summary of the mathematical order of parameters and terms using various formulae for vectorial capacity. Example interventions are given for each parameter

Reference	Vectorial capacity, *V*	$\epsilon_{V}(m_{0})$ (Introduction of a refractory gene in the adult mosquito population)	$\epsilon_{V}(\lambda_{0})$ (Larval source management)	$\epsilon_{V}(G_{0})$ (Delayed blood feeding or increased adult mortality)	$\epsilon_{V}(n_{0})$ (Bacterial symbiont that delays parasite maturation)	$\epsilon_{V}(Q_{0})$ (Non-insecticide treated bednets)	$\epsilon_{V}(\;f_{0})$ (Barrier methods targeted to all hosts)	$\epsilon_{V}(g_{0})$ (Insecticide-based methods)
Ross-Macdonald^10^	$\frac{ma^{2}p^{n}}{-\text{ln}(\;p)}$	1	NA	NA	*gn*	2	2	1+*gn*
Smith and McKenzie^22^	$\lambda \frac{\;f^{2}Q^{2}e^{-gn}}{g^{2}}$	1	1	NA	*gn*	2	2	2+*gn*
Current analysis	$\lambda (G)S^{2}P$	1	1	*ϕ*	*gn*	2	2+*ϕ*	2+*ϕ*+*gn*

NA: Not applicable.

### The mathematical order of population dynamic feedbacks

The analysis so far describes sensitivity of vectorial capacity without considering the effects of juvenile population feedback. In the mathematical model defined above, it is possible to evaluate the sensitivity of mosquito population density (*m*) to adult vector control through its effects on the reduced emergence rate from the juvenile population (*λ*) in the next generation. The equation for juvenile dynamics (see Materials and methods) suggests that egg laying, which is affected by the adult traits of mosquito survival and blood feeding, could have an extra effect on vectorial capacity. To integrate this analysis into Macdonald's, we must ask: ‘what is the order of a change in these parameters on the rate of emergence of adult mosquitoes?’ What is *ϕ*? such that:

$$\epsilon_{\lambda }(G_{0})=\phi .$$

The class of models that we have described in the Materials and methods section includes habitat heterogeneity, density independent mortality and density-dependent mortality that obeys a power-law response to mean crowding.^13,23,28^ Analysis of such models suggests the answer depends on migration of mosquitoes, the importance of density-independent versus density-dependent aspects of juvenile mosquito ecology and the robustness of local population dynamics, the interaction of which is explored in the Supplementary information.

The full system in the heterogeneous open population simulation model is sufficiently complicated that results of analysis are difficult to interpret, but it is possible to develop some useful insights by examining progressively more complicated models and the environments that they represent.

#### Homogeneous, closed populations

In the simple case when there is no mosquito migration and no local habitat heterogeneity then elasticity would be a simple function of eggs laid (Supplementary information):

$$\epsilon_{\lambda }(G_{0})=-\frac{G_{0}}{G_{0}-\tau }\;,$$

where τ is the threshold number of eggs laid per female required for population persistence. The elasticity of *G*_0_ (and by extension, the added elasticity of *f* and 1/*g* for vectorial capacity) is extremely high near values that describe thresholds for mosquito population persistence (i.e. $G_{0}\approx \tau$ but $G_{0}>\tau$). If at baseline $G_{0}>\tau$, but $G_{C}<\tau$ after control, i.e. elimination, then the effect size would be infinite and elasticity undefined. In robust populations, where egg laying far exceeds the threshold for population persistence, the elasticity tends to be close to 1. In other words, *G* has a 1^st^ order effect in mosquito populations with no migration and robustly stable internal dynamics.

#### Homogeneous, open populations

**Figure 1.**
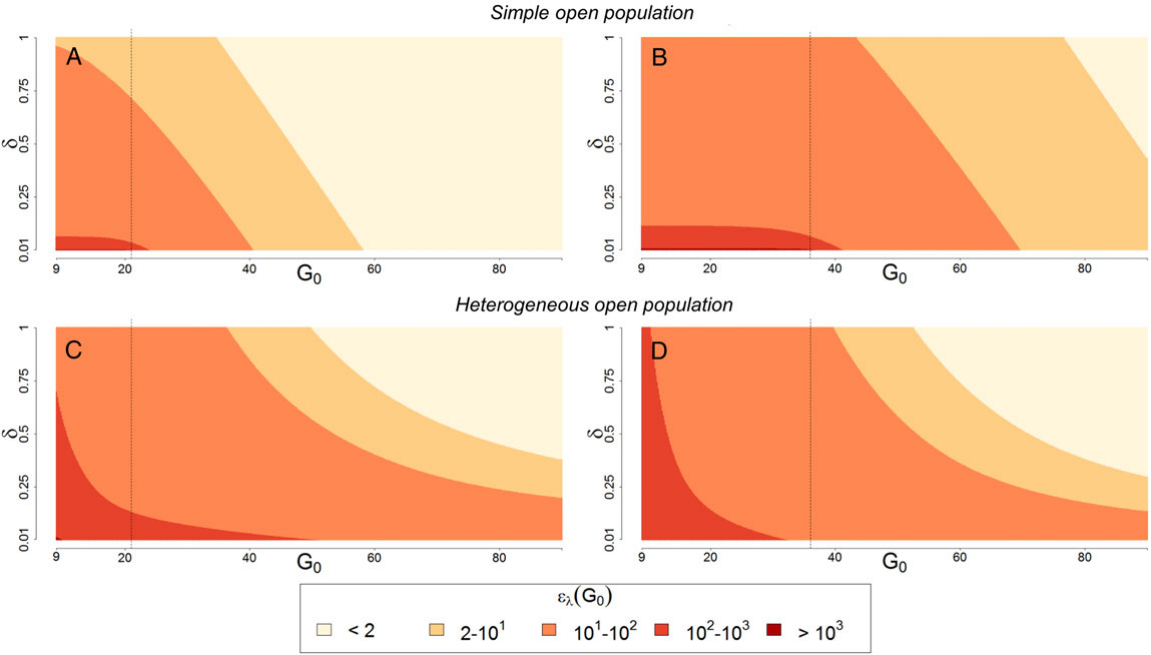
Heat plots of the elasticity of *G*_0_, i.e. the expected order of effect changes in *G*_0_ will have on vectorial capacity, at different baseline values of *δ*, *G*_0_ and *τ* for steady state open mosquito populations. The upper panel (A and B) show the effects in a simple open population using the equation described in the Supplementary information. The lower panel (C and D) shows the effects in a heterogeneous open population using the simulation model described in the Materials and methods section. Left (A and C) and right (B and D) panels show the results for different values of the population persistence threshold parameter (*τ*), which is shown by a black dotted line.

Although it is possible to develop a mathematical formula describing $\epsilon_{\lambda }(G_{0})$ in simple, open populations, it is difficult to interpret. Using the formula presented in the Supplementary information, however, it is clear that effect sizes depend on the ratio λ/δ, as well as the egg-laying threshold for population persistence *τ* (Figures 1A–B). The main difference between open and closed populations is that the local mosquito population always persists because of immigration.

Effect sizes in open populations are similar to the effect sizes in closed populations when immigration is low relative to local recruitment (i.e. if δ≪λ, Figures 1A–B). The interpretation of effect sizes changes, however, when the rate of immigration is approximately equal to or higher than local productivity. In these cases, vectorial capacity is reasonably unaffected by any population dynamic feedbacks because mosquito population density is determined by immigration and is relatively insensitive to changes in egg laying. In the extreme case, local mosquito habitat could be a demographic sink for mosquito populations. Immigration does, however, limit the total reduction in local mosquito population density that can be achieved through control. Ignoring population dynamic feedbacks, adult mosquito population density would still be linearly affected by changes in mortality, but it is comparatively unaffected by changes in feeding rates. In summary, if LSM were attempted in a small area, the elasticity could be less than 1, but it would still approach 1 if *G*_0_ was much larger than *τ*.

#### Heterogeneous, open populations

In the general analysis, with habitat heterogeneity and lifetime egg-laying rates that are much larger than the local persistence threshold for mosquito populations (Supplementary information and Figures 1C–D) the elasticity of egg-laying is approximately equal to 1:

$$\epsilon_{\lambda }(G_{0})\approx 1.$$

In models with low rates of mosquito immigration, elasticities behave similarly. As the rate of mosquito immigration is set to progressively higher numbers, the sensitivity of mosquito population dynamics to local control declines (Figures 1C–D).

Although habitat heterogeneity changes the shape of the surface describing elasticity of egg-laying as a function of baseline egg-laying, thresholds for persistence, and immigration, all of the former results still hold (Figure 1). Our elasticity analysis thus suggests that, in models of this type and in the ecological situations they mimic, delaying blood feeding or increasing adult mortality would reduce net emergence rates approximately linearly.

#### Elasticity revisited

**Figure 2.**
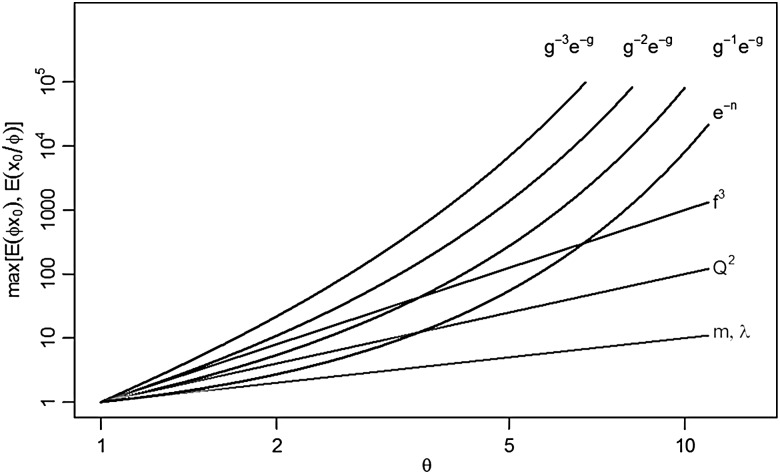
The revised overall picture of logged effect sizes (y axis) for up to 90% reduction in mosquito bionomic parameters plotted against logged proportional changes in the underlying parameters (*θ*, x axis). These follow from the three simple rules stated in the text for determining the shape of elasticity relationships for parameters that enter the formula for vectorial capacity in different ways (see table 2).

In light of these population-dynamic feedbacks, Macdonald's original logic can be revisited through the evolving formulae for vectorial capacity. Elasticities of parameters in our expanded definition of vectorial capacity have a clear ranking, which can be illustrated by looking at the effects of halving the value of a parameter (Table 2). Changes in mosquito population density (or the frequency of a gene that makes mosquitoes refractory to infection) would have linear (1^st^ order) effects on vectorial capacity, so halving mosquito population density halves transmission (i.e. to 50% of baseline). Elasticity of the EIP (assuming ng≈ 1) would have a 1^st^ order effect, but doubling EIP more than halves transmission (i.e. by 63%). Human feeding proportions have order 2, so diverting half the bites onto non-human hosts reduces transmission by a factor of 2^2^=4 (i.e. by 75%); with a 1^st^ order feedback, blood feeding rates have order of approximately 3, so halving feeding rates reduces transmission by a factor of 2^3^=8 (i.e. by 87.5%). Finally, adult mosquito mortality has order 3+ng≈ 4 so halving mosquito lifespan would cut transmission by more than a factor of 2^4^=16 (i.e. by 93.75%). Larger changes in parameters affecting survival through EIP would have a larger effect (Figure 2).

## Discussion

Here we have shown that Macdonald's original analysis, and those that have followed, underestimated the sensitivity of transmission to overall blood feeding rates and to mosquito mortality rates because they did not take into account adult-juvenile population feedbacks. Vector-based interventions can increase mosquito mortality, increase the interval between blood meals, divert some bites onto non-human hosts, or reduce the productivity of larval habitats. The consequences of these changes on transmission can be interpreted using the formula for vectorial capacity (Table 2). Macdonald used such arguments to explore the reasons for the success of DDT spraying programs. He argued that DDT reduced survival of adult mosquitoes, and survival affected transmission in two ways: 1) a reduced number of mosquito bites, 2) reduced survival through the EIP.^10,12^ This analysis helped explain why indoor spraying with DDT worked so well in early field trials, justified expansion of the programs for malaria eradication^11,12^ and had a long and profound influence on policy for malaria prevention.^29^ Later, Macdonald's model was reformulated to follow cohorts of mosquitoes from birth to show that increasing mortality would also reduce adult mosquito population density (Second row of Table 2,^22^). Here, reanalysis of these formulae suggests that, by reducing egg-laying, adult vector control will add another effect on malaria transmission not previously considered through additional feedbacks to mosquito population density (Third row of Table 2).

The importance of these effects will, however, depend on the baseline state of local mosquito population dynamics. One useful way to understand this is the spatial scales at which vector control is implemented relative to the spatial scales at which mosquito populations are connected. The models suggest that effective adult control will either need to account for the additional efforts needed to control immigration from external populations, or occur at sufficient spatial scale as to minimise the influence of immigration relative to internal dynamics. Experimental quantification of adult mosquito immigration is, therefore, an important factor to consider with reference to the spatial scales of control.

It is also important to consider these results in light of various model assumptions. Like many mosquito transmission models we assume homogeneous mixing of vectors and hosts. This may be particularly relevant to mosquito immigration and emigration (and control efforts targeting migration) that may be spatially structured.^28^ In this analysis we assume density dependent-mortality follows a power-law distribution, which is supported by experimental evidence for lower, more realistic density levels.^25^ Density-dependent processes can be ecologically complex and extensions could investigate other effects of increased density, such as population-dependent immigration compensation or emergence time delays with explicit cohorts of juvenile mosquito life-cycle stages.^15,30^ Finally, these models evaluate mosquito population dynamics at equilibrium, despite many species exhibiting seasonal variations in abundance. Other expressions of vectorial capacity are available to evaluate seasonally varying dynamics^31^ and could be developed to reassess the conclusions presented here, albeit with considerably more complexity.

Macdonald's post-hoc analysis helped justify the use of DDT for malaria eradication, but any attempt to apply Macdonald's analysis beyond this original purpose calls into question the conclusion that vector control should always prefer methods that attack adult mosquitoes over methods that attack juvenile populations in aquatic habitats.^32^ A recent analysis of LSM exposed the limitations of universal application of Macdonald's original analysis and its over-reliance on the concept of mathematical sensitivity to parameters.^13^ Recent analysis has explicitly considered mosquito population dynamics and LSM. Although the models generally concur that the emergence rate of adult mosquitoes has a linear effect on mosquito density, they also suggest mosquito density could respond in a highly non-linear way to intervention coverage.^13^ The prospects for success, including operational concerns, would depend on other aspects of the ecology. The recent analysis shows that LSM is an important component of transmission that was intentionally disregarded in Macdonald's analysis, which was always suspect with respect to LSM because the formulae for vectorial capacity did not convey any information about juvenile mosquito populations or population dynamics.^24^ A secondary result suggested by these formulae, and meriting further exploration, is that since LSM would be expected to raise the threshold on egg-laying for local population persistence through increased mortality in aquatic habitats, adult vector control could have even greater effects when paired with LSM, especially if targeted application can lead to high coverage.

In any case, the proper basis for comparing vector-based interventions is not the mathematical order per se, but the overall reductions in transmission and the burden of disease that would come from reaching coverage levels with different interventions at comparable costs. Indeed, available evidence suggests that LSM achieves comparable reductions in transmission for comparable costs.^32^ Reaching a certain policy objective in situations with either high baseline transmission intensity or refractory vectors may not be possible using a single mode of vector control. Achieving a policy objective might require integrated vector control, which could involve attacking various vector species in different ways, or achieving very high coverage levels with multiple interventions. The analysis described herein provides a basis for understanding and predicting how those interventions would affect transmission when combined, although ultimately effectiveness should be evaluated using field trial data.

Sensitivity or elasticity analyses are useful ways of understanding how to translate measurable changes in mosquito populations brought about through control into useful information about transmission within a given model. Arguments about sensitivity to parameters provide useful rules of thumb, but such rules should be used with caution, because malaria transmission often occurs in complex transmission settings with dominant and minor vectors in heterogeneous populations.^33^ In policy settings, it is critically important to determine whether the interventions achieve the overall goal of reducing the burden of or eliminating malaria. These require an understanding of the ways that changes in coverage of various interventions alone and in combination translate into changes in the underlying parameters of mosquito and parasite populations, and on the overall coverage levels that are achievable in various settings. These broader concerns, more than sensitivity to parameters, should dictate how vector control programs are designed, tailored to context and evaluated.

## Supplementary data

Supplementary data are available at *International Health* online (http://inthealth.oxfordjournals.org/).

Supplementary Data
