# Supplementary material for: Adult vector control, mosquito ecology and malaria transmission
Source: Int Health. 2015 Feb 26;7(2):121–9. doi: 10.1093/inthealth/ihv010 (PMC4357799; doi:10.1093/inthealth/ihv010)
Supplement: Supplementary Data [file supp_7_2_121__index.html]

Supplementary Data 

# Adult vector control, mosquito ecology and malaria transmission

## Supplementary Data

Supplementary Data

**Files in this Data Supplement:**

- Supplementary Figure 1 - pdf file
- Supplementary data - docx file
