# Supplementary figures and images for: Adult vector control, mosquito ecology and malaria transmission
Source: Int Health. 2015 Feb 26;7(2):121–9. doi: 10.1093/inthealth/ihv010 (PMC4357799; doi:10.1093/inthealth/ihv010)

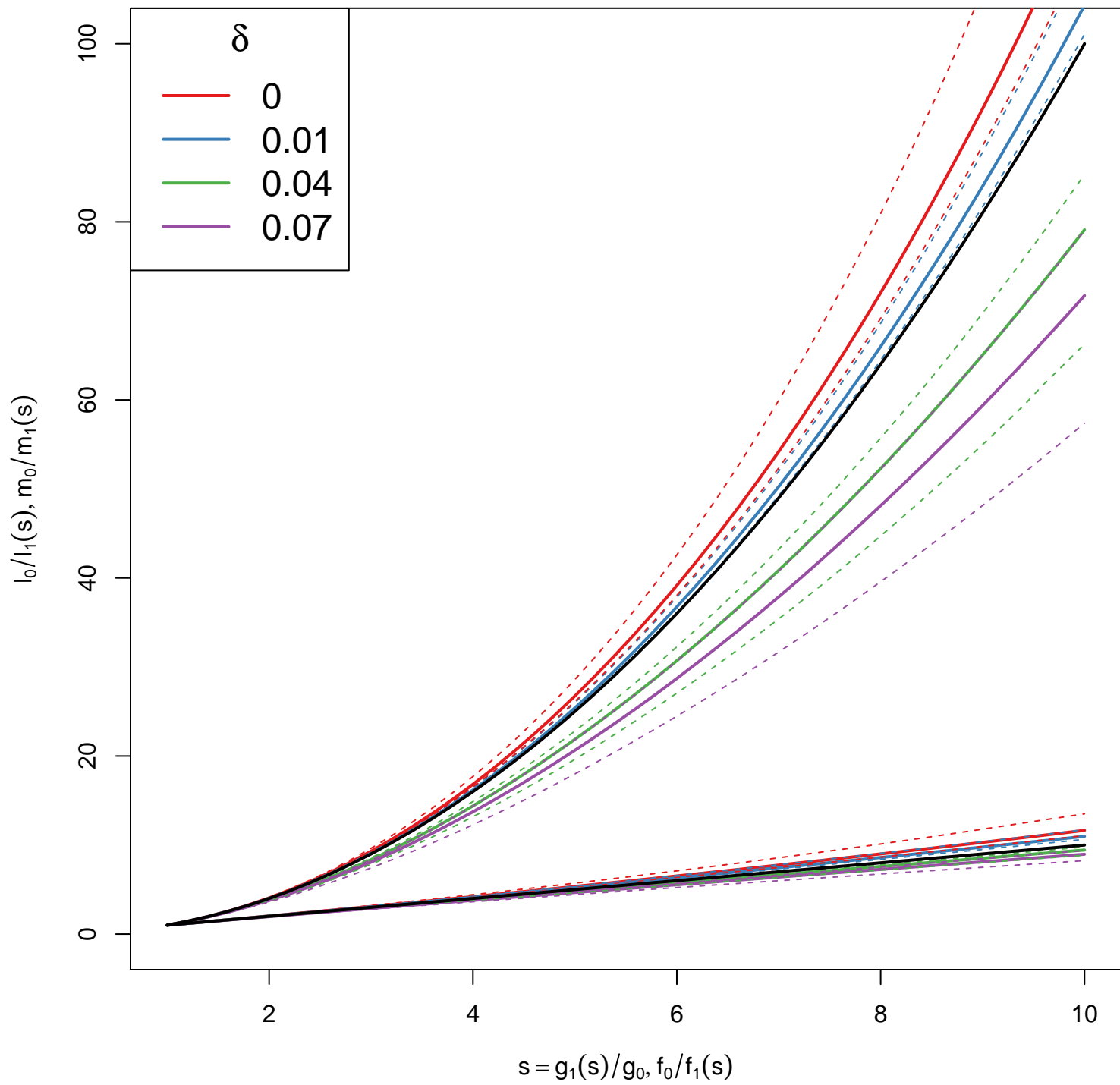

Supplement: Supplementary Data [file supp_ihv010_ihv010supp_fig1.pdf]
