# Supplementary material for: Adult vector control, mosquito ecology and malaria transmission
Source: Int Health. 2015 Feb 26;7(2):121–9. doi: 10.1093/inthealth/ihv010 (PMC4357799; doi:10.1093/inthealth/ihv010)
Supplement: Supplementary Data [file supp_ihv010_ihv010supp_data.docx]

**Supplementary information**

**Simple, closed population:** $\delta=0;\omega=0;N=1;\sigma=1$

In this case at the steady state,

$$\lambda=\alpha l=gm=\left( \frac{fv}{g}-\frac{\gamma}{\alpha}-1 \right)\frac{\alpha^{2}}{\psi}=\left( G-\frac{\gamma}{\alpha}-1 \right)\frac{\alpha^{2}}{\psi}.$$

Note that a threshold condition for persistence of the mosquito populations is:

$$G=\frac{fv}{g}>1+\frac{\gamma}{\alpha}=\tau.$$

We note both $f$ and $1/g$ have a linear effect on the total number of eggs laid over the mosquito lifespan, therefore they will have the same elasticity, but with a different sign. The effect size associated with $G$ is thus:

$$E_{\lambda}\left( G | G_{0} \right)=\frac{G_{0}-\tau}{G_{C}-\tau}.$$

The elasticity of $G$ is:

$$\varepsilon_{\lambda}\left( G_{0} \right)=-\frac{G_{0}}{G_{0}-\tau}.$$

If the mosquito population is near its threshold for persistence, then the elasticity exceeds 1 by a very large margin. As the egg-laying leads to increasingly crowded conditions, the elasticity declines, but it is always greater than 1.

**Simple, open population:** $N=1;\sigma=1;\omega=0;\delta>0$

When there is some immigration of mosquitoes from outside an area of interest, the dynamics change. As long as$\delta>0$, the mosquito population persists, and the steady state productivity is:

$$0=G\delta+\left( G-1-\frac{\gamma}{\alpha} \right)\lambda-\frac{\psi}{\alpha^{2}}\lambda^{2},$$

Where $\tau$ is now a local threshold and $\tau=1+\gamma/\alpha$, as before, which is a population persistence threshold on egg-laying that would apply if there had been no migration. If there is some migration, then the population always persists, but the threshold is still a relevant parameter:

$$\lambda=\frac{\left( G-\tau\right)+\sqrt{{(G-\tau)}^{2}+4G\delta\frac{\psi}{\alpha^{2}}}}{2\frac{\psi}{\alpha^{2}}}.$$

The effect size associated with $G$ is:

$$E_{\lambda}\left( G_{C} | G_{0} \right)=\frac{\left( G_{0}-\tau\right)+\sqrt{{(G_{0}-\tau)}^{2}+4G_{0}\frac{\delta\psi}{\alpha^{2}}}}{\left( G_{C}-\tau\right)+\sqrt{{(G_{C}-\tau)}^{2}+4G_{C}\frac{\delta\psi}{\alpha^{2}}}}.$$

and the elasticity is:

$$\varepsilon_{\lambda}\left( G_{0} \right)=\frac{G_{0}\left( 1+\frac{G_{0}+4\frac{\delta\psi}{\alpha^{2}}}{\sqrt{\left( G_{0}-\tau\right)^{2}+4G_{0}\frac{\delta\psi}{\alpha^{2}}}} \right)}{\left( G_{0}-\tau\right)+\sqrt{{(G_{0}-\tau)}^{2}+4G_{0}\frac{\delta\psi}{\alpha^{2}}}}.$$

Plotting elasticity (Figures 1A and 1B, main manuscript) shows that as $G_{0}$ becomes large, the elasticity of $G$ approaches 1. If $\delta$ is relatively small and $G_{0}$ close to or below the would-be threshold for persistence for the closed population, elasticity tends to far exceed 1 in a similar manner to the simple closed population. By progressively increasing $\delta$ (and holding $\alpha^{2}/\psi$ fixed), the elasticity reduces towards 1 under every value of $G_{0}$. The elasticity-reducing effect of increasing $\delta$ is particularly apparent the further $G_{0}$ exceeds $\tau$ (Figures 1A and 1B, main manuscript). Under every value of $\delta$, elasticities approach 1 as $G_{0}$ becomes large (Figures 1A and 1B, main manuscript).

**General case:**

In environments with heterogeneous aquatic habitats, model parameters are variable and the effect sizes of certain parameters cannot be simply evaluated using a first principles approach. Instead the following simulation was implemented using realistic parameter value ranges based on Smith et al.^1^ applied to a set of $i$ pools (Supplementary table 1). The functional forms are based on standard functions to draw random deviates in R version 3.1.0 (R Development Core Team, Vienna, Austria).

**Supplementary table 1.** Parameter values for the heterogeneous open population simulation

| Parameter | Description | Simulated values |
| --- | --- | --- |
| $f$ | Adult female mosquito blood feeding rate | $0.3 d^{-1}$ |
| $g$ | Instantaneous adult mosquito death rate | $0.083-0.83 d^{-1}$ |
| $v$ | Number of eggs laid per blood feed | $25$ |
| $N$ | Number of aquatic habitats | $30$ |
| $p_{i}$ | Proportion of eggs laid in the $i^{th}$ pool | $rBeta(1,10)$ |
| $\alpha_{i}$ | Adult mosquito emergence rate of the $i^{th}$ pool | $0.1 d^{-1}$ |
| $\gamma_{i}$ | Density independent mortality rate of the $i^{th}$ pool | $rBeta(1,9)$ |
| $\Psi_{i}$ | Per-capita mortality increase of the $i^{th}$ pool due to crowding | $rBeta(1,19)$ |
| $\sigma_{i}$ | Order of the effects of density-dependence of the $i^{th}$ pool | $1$ |
| $\delta$ | Immigration rate of adult female mosquitoes | $0.01-1$ |

Note that while we only change model input values of $g$, the effect on the elasticity of $G_{0}$ is analogous to changing $f$ as both appear ina linear form in the equation for $G$, thus the model can explore the elasticity of increased mosquito mortality and/or delayed blood feeding.

For this simulation we set $\sigma_{i}=1$ which is representative of the classic first order description of density-dependence as described by the logistic growth equation.

Immigration was set as a proportion ($\delta$) of the sum of the carrying capacity ($K$) for all aquatic habitats, where:

$$K=\frac{fv\alpha_{i}-g(\alpha_{i}+\gamma_{i})}{g\psi_{i}}.$$

We evaluated the elasticity of $G$ with respect to different values of $\delta$, $G_{0}$ and $\tau$ to test if the generalisations in models of simple populations held true for more complex models of heterogeneous populations. Default values from Supplementary table 1 were used. To fix the value of $\tau$, the random deviates of $p$ were scaled proportionally.

To decrease processing time for each simulation, the elasticity of $G$ was calculated for combinations of just ten different values of $\delta$ and $G_{0}$ (giving 100 different values of $\varepsilon(G)$). Regression spline generalised additive models with a Gaussian link function were used to predict intermediate values with 1000 predictions each of $\delta$ and $G_{0}$, giving a total of 1000^2^ values of $\varepsilon_{\lambda}\left( G_{0} \right)$. Generalised additive models were fitted using the ‘mgcv’ package and the equilibrium of the population dynamic equations were evaluated with the ‘deSolve’ package in R version 3.1.0. The mean of 100 simulations was taken to give the results in Figures 1C and 1D (main manuscript)

The results of this heterogeneous population model simulation are consistent with those from simple open populations; as both $G_{0}$ and $\delta$ increase, $\varepsilon_{\lambda}\left( G_{0} \right)$ tends towards 1 (Figures 1C and D, main manuscript). In contrast to the simple open population $\varepsilon_{\lambda}\left( G_{0} \right)$ is much less sensitive to changes in either $G_{0}$ or $\delta$, particularly if the other parameter is minimal (contrast Figures 1A and B with C and D, main manuscript).

To quantify uncertainty in our predictions of $\varepsilon_{\lambda}\left( G_{0} \right)$ we performed 1000 runs with random parameter draws from Supplementary table 1 for selected values of $G_{0}$ and $\delta$. The 10^th^ and 90^th^ percentile of relative adult and juvenile population densities were calculated and are shown in Supplementary Figure 1.

**References**

1. Smith DL, Perkins TA, Tusting LS et al. Mosquito population regulation and larval source management in heterogeneous environments. PLoS One 2013;8:e71247.

**Figure legend:**

**Supplementary figure 1.** The order of effect size of changes in adult mortality $(g)$ on equilibrium adult (top lines) and juvenile (bottom lines) mosquito populations in the presence of varying levels of immigration $(\delta)$. For small amounts of immigration the response of the adult population is approximately quadratic (black line $s=s^{2}$) which corresponds to 1) a first order effect of reduced adult population density and 2) an approximately first order effect of reduced adult density due the reduced number of individuals emerging from juvenile habitats. The equivalent response of the juvenile population is approximately linear (black line $s=s$) reflecting just the adult juvenile feedbacks. As immigration increases the effect size of adult mortality on adult mosquito population size linearizes as adult-juvenile feedbacks have a reduced effect on adult population size. In this figure the median (solid lines) and 10–90^th^ percentile (dotted lines) of predictions from the 1000 model runs for four different levels of immigration are shown. Lines closer to the black $s=s^{2}$ line show changes in the adult population, whereas lines clustered around the black $s=s$ line show changes in the juvenile population.
